# Burnout Syndrome Predictors in Nursing Professionals During and After the COVID‐19 Pandemic: A Prospective Cohort

**DOI:** 10.1111/jocn.70216

**Published:** 2026-01-17

**Authors:** Miguel Lucas Silva da Paixão, Daiane Dal Pai, Tânia Solange Bosi de Souza Magnago, Fábio Fernandes Dantas Filho, Silvia Cristina Garcia Carvalho, Gabriel Fernandes Gonçalves, Luciana Olino, Juliana Petri Tavares

**Affiliations:** ^1^ Federal University of Rio Grande do Sul Porto Alegre Rio Grande do Sul Brazil; ^2^ Federal University of Santa Maria Santa Maria Rio Grande do Sul Brazil

**Keywords:** burnout syndrome, cohort studies, COVID‐19, nursing

## Abstract

**Aim:**

To analyse predictors of burnout in nursing professionals during and after the COVID‐19 pandemic.

**Design:**

Cohort study.

**Method:**

A two‐phase study conducted during the COVID‐19 pandemic's peak (2020) and post‐vaccination period (2022). Data from nursing professionals of four hospitals in southern Brazil included sociodemographic, occupational, lifestyle, and health variables, and Maslach Burnout Inventory responses. Multivariate logistic and linear regression analyses were used to identify independent predictors of burnout syndrome. The study was approved by the Research Ethics Committee (approval no. 4.152.027).

**Results:**

A sample of 163 participants were assessed at two distinct time points. In 2020, 9.2% of nursing professionals experienced burnout syndrome, decreasing slightly to 7.4% in 2022. As for the burnout dimensions, emotional exhaustion was reported by 27% of professionals in 2020 and 26.4% in 2022. Depersonalisation affected 28.2% during the pandemic and 25.2% afterward. Low professional accomplishment was identified in 29.4% of professionals in 2020, increasing to 30.1% in 2022. Distinct predictors were identified for overall burnout and its specific dimensions. The main predictors included: perceiving a mental health impact from the pandemic, previous mental health issues, recent medical leave, and working directly with COVID‐19 patients.

**Conclusions:**

Burnout syndrome remained stable post‐pandemic. Key predictors were identified, highlighting the need for preventive mental health interventions.

**Relevance for Clinical Practice:**

Identifying predictors of burnout in nursing professionals supports the development of targeted interventions to protect mental health, improve job satisfaction, and enhance the quality of patient care during and after health crises.

**Impacts:**

This study fills a gap in post‐pandemic research by identifying predictors of burnout in nursing professionals. It supports the development of policies and interventions to protect mental health and improve working conditions in Brazilian hospitals.

**Reporting Method:**

STROBE guidelines for cohort studies.

**Patient or Public Contribution:**

Participants contributed only through data collection.

## Key Points

- Perceived mental health impact of the COVID‐19 pandemic and pre‐existing psychiatric disorders were consistent predictors of burnout across periods.
- Providing direct care to COVID‐19 patients predicted burnout during the pandemic, while fear of infection, medical leave, and managerial roles emerged as post‐pandemic predictors.
- Burnout syndrome prevalence among nursing professionals remained stable between the pandemic peak (2020) and the post‐pandemic period (2022), despite changes in work context.
- Emotional exhaustion, depersonalisation, and low professional accomplishment affected approximately one‐quarter to one‐third of professionals at both time points.
- Absenteeism due to non‐COVID‐19 medical leave was strongly associated with burnout, highlighting the interplay between health deterioration and work overload.

## 1 Introduction

Burnout syndrome (BS) primarily affects professionals whose work involves interpersonal relationships, such as healthcare workers—particularly members of the nursing staff (Lautert 1995). This is due to their continuous presence at the bedside, where they face stressful and challenging situations while caring for patients, including heavy workloads, overburden, and occupational stress. Moreover, many need a second job, and resort to caffeinated beverages and stimulant medications to compensate for a lack of adequate sleep and rest, thereby increasing their risk of developing negative mental health conditions (Lautert 1995; Soares et al. 2021).

BS is characterised by three core components: emotional exhaustion, depersonalisation, and reduced professional accomplishment. Emotional exhaustion refers to a feeling of being overwhelmed or overworked. Depersonalisation relates to a sense of detachment or feeling disconnected from one's work, often characterised by impersonal attitudes and a lack of empathy toward patients. Low professional accomplishment is associated with reduced feelings of efficacy and professional competence (Maslach and Jackson 1981).

As a consequence, this context significantly impacts workers' health. Internationally, high rates of this condition are observed among nursing professionals, prompting discussion and concern. A meta‐analysis of studies from thirty countries conducted between 2012 and 2022 found a global prevalence of 30% for burnout symptoms among nursing staff (Rizzo et al. 2023). In Brazil, poor working conditions, mismanagement within the healthcare system, and frequent shortages of essential equipment commonly exacerbate psychological pressure and physical exhaustion (Cardoso et al. 2021).

It is well known that professionals facing such adversities may experience negative impacts on the quality of care provided, impairing their ability to carry out responsibilities effectively, compromising clinical judgement and decision‐making (Simonetti et al. 2021).

During the pandemic, healthcare professionals' mental health was further compromised due to the high demand for care and rapidly changing work protocols. The severity of the situation rendered these workers even more vulnerable to the development of burnout‐related symptoms (Wang et al. 2024).

Healthcare workers whose physical and mental health were already compromised showed a significantly higher incidence of errors compared to their peers, indicating a direct correlation between psychological and physical distress leading to suboptimal patient care, ultimately jeopardising patient safety (Melnyk et al. 2021).

In early 2021 COVID‐19 vaccination began, leading to reductions in infection and mortality rates, and alleviating the overcrowding in intensive care units and other healthcare services. With the epidemiological situation under control, the World Health Organization declared the end of the COVID‐19 pandemic in 2023 (PAHO 2023). Nevertheless, the consequences of such a healthcare crisis are expected to persist for an extended period, referred to as the “post‐pandemic” phase (Peters et al. 2022). This projection stems from the recognition that COVID‐19 exposed significant flaws and vulnerabilities in current systems, highlighting the need for resilience, equity, diversity, compassion, and inclusive policies (Leach et al. 2020).

The implementation of changes in institutions' work environment can significantly contribute to strengthening organisational and occupational resilience. Resilience is a key element in coping with the continuous transformations in the work context, particularly regarding the safety, health, and well‐being of professionals during and after a health crisis. Therefore, it is essential to investigate the post‐pandemic period and its impacts on workers' health (Peters et al. 2022).

Although prospective cohort studies are suitable to elucidate longitudinal factors related to burnout among nursing professionals, no such studies have been identified in Brazilian literature during and after the pandemic. There remains a knowledge gap regarding BS among nursing professionals in the post‐pandemic period, as the scientific literature has primarily focused on analysing workers' mental health during the pandemic (Galanis et al. 2021; Peters et al. 2022; Woo et al. 2020; Leach et al. 2020).

Based on the aforementioned considerations, this study aims to address the following research question: Did the occurrence of BS among hospital‐based nursing professionals decrease after the COVID‐19 pandemic? The hypothesis is that nursing professionals present lower levels of BS after the pandemic. The aim of this study is to analyse predictors of burnout in nursing professionals during and after the COVID‐19 pandemic.

This project is part of a broader research initiative entitled “Work during the COVID‐19 Pandemic: impacts on the Mental Health of Nursing Workers” approved by the Research Ethics Committee under opinion number 4.152.027.

## 2 Method

The present project is part of a larger research entitled “Work during the COVID‐19 Pandemic: impacts on the Mental Health of Nursing Workers”, approved under protocol number 4.152.027.

This study was conducted in accordance with the guidelines of the Strengthening the Reporting of Observational Studies in Epidemiology (STROBE), which provides a checklist with recommendations for observational studies (Malta et al. 2010). The following are the steps comprising this study.

### 2.1 Study Design

This is a multicenter, quantitative, analytical, and prospective cohort study carried out in two phases: the first in 2020 and the second in 2022. The same group of nurses who participated in the first phase were followed and reassessed during the second phase, allowing for a longitudinal analysis of changes over time. This design aligns with the principles of prospective cohort studies, in which participants are enrolled before the outcomes of interest occur and are observed over a defined period. Unlike cross‐sectional studies, the prospective cohort approach enabled us to measure the incidence and progression of specific outcomes within the same population, thereby offering a more robust understanding of temporal changes and potential causal relationships.

### 2.2 Study Setting

The study was conducted in four tertiary hospitals, all of which are leading healthcare facilities within the Brazilian Unified Health System (SUS), and had its service modified to treat patients affected by COVID‐19 in the state of Rio Grande do Sul.

The institutions are referred to as Hospital A (HA), Hospital B (HB), Hospital C (HC), and Hospital D (HD) to minimise exposure and preserve anonymity. HA is a public, general, and teaching hospital with 784 available beds. HB is also a public and a teaching hospital, recognised as a trauma care reference center, with 237 beds. HC is a public, general, and university hospital with a capacity of 850 beds. Finally, HD is a public, general, and university unit with 403 beds.

### 2.3 Participants

The study population consisted of 6.899 nursing professionals from the four hospitals: 2.962 from HA, 707 from HB, 2.278 from HC, and 952 from HD. The data was provided by the institutions in 2020.

Included in the study were nursing professionals who worked on in‐patient care during the COVID‐19 pandemic, and answered the data collection forms during the pandemic's peak (Phase 1, year 2020) as well as during post vaccine phase (Phase 2, year 2022). Excluded were workers who had been on leave for more than 30 consecutive days during the period between the first and second phases of data collection.

### 2.4 Variables, Measures, and Data Sources

Burnout syndrome (BS) was assessed using the Maslach Burnout Inventory (MBI) (Maslach and Jackson 1981), with a brazilian validated version (Pereira 2015), composed of 22 Likert‐scale questions ranging from 0 to 5 points. Nine questions assess emotional exhaustion (items 1, 2, 3, 6, 8, 13, 14, 16, and 20), five assess depersonalisation (items 5, 10, 11, 15, and 22), and eight assess reduced personal accomplishment (items 4, 7, 9, 12, 17, 18, 19, and 21), with this last domain being scored inversely. The syndrome is only diagnosed when all three domains have high scores, assessed using the interquartile ranges found in the studied sample.

Sociodemographic (sex, age, race/ethnicity, marital status, and education level), lifestyle and health (smoking, alcohol consumption, physical activity, and medication initiated during the pandemic), and occupational data (workplace, years in the profession and at the institution, job position, type of employment contract, work at another institution, managerial role, work shift, reassignment during the pandemic, patient care for individuals with COVID‐19 and perceived fear regarding the risk of contamination) were collected.

Data collection was based on the database established by the larger project, composed of sociodemographic variables and the selected research instrument.

### 2.5 Bias

As a prospective cohort study, the results may be influenced by confounding variables that affect the association between exposure and outcome. To control for these effects, Poisson regression with robust variance was used, as it allows for the estimation of adjusted prevalence ratios and the independent effect of each variable on the outcome. This approach is appropriate for studies with categorical outcomes and offers a valid alternative to logistic regression when the goal is to estimate prevalence ratios rather than odds ratios (Barros and Hirakata 2003). The variables included in the regression models were selected based on theoretical relevance and bivariate analysis (*p* < 0.05).

### 2.6 Study Size

Sample size was estimated using the Power and Sample Size for Health Researchers software (v0.18), assuming a prevalence of 12% (de Magalhães et al. 2022), 80% power, and a 5% significance level. The minimum required sample was 150 participants.

**FIGURE 1:**
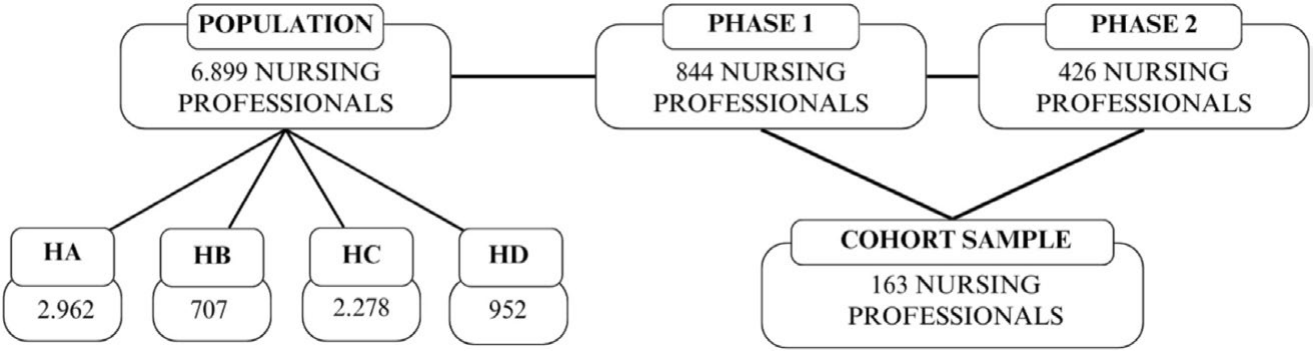
Participant selection and follow‐up flowchart. Porto Alegre, 2025.

In the first phase (2020), 844 nursing workers (nurses, technicians, and assistants) completed the survey. In the second phase of data collection (2022), 426 nurses completed the survey. Of these, 163 participants were identified as having also responded in the first phase (2020), as shown in Figure 1. As no unique personal identifiers were collected in order to preserve participant anonymity, date of birth was used as a variable to enable record linkage across waves, allowing identification and description of participant retention over time.

Participant loss was expected due to the nature of cohort studies and high turnover during the COVID‐19 pandemic, including temporary contracts and staff reassignments. The final sample exceeded the estimated minimum and preserved statistical power.

### 2.7 Quantitative Variables and Data Analysis

Information was collected from the structured database in both phases of the study, including sociodemographic characteristics and burnout assessment using the Maslach Burnout Inventory (MBI) (Maslach and Jackson 1981), open‐access version (Pereira 2015).

Data were entered into Excel and analysed using Statistical Package for the Social Sciences (SPSS) v.18. Normality was assessed using the Shapiro–Wilk test. Continuous variables were described using measures of central tendency and dispersion; categorical variables were reported as absolute and relative frequencies. Associations between categorical variables were tested using the Chi‐square or Fisher's exact test, and McNemar's test was applied to paired nominal data.

Prior to multivariate linear regression modelling, standard diagnostic procedures were performed to assess whether the underlying statistical assumptions were adequately met. Linearity between independent variables and outcomes was evaluated through visual inspection of residuals versus fitted values. The normality of residuals was assessed using histograms and Q–Q plots. Homoscedasticity was examined through residual scatterplots, revealing no systematic patterns. Independence of observations was ensured by the study design, and multicollinearity was evaluated using variance inflation factors. For multivariate analysis, Poisson regression with robust variance was applied to estimate adjusted prevalence ratios for categorical outcomes. Logistic regression (Stepwise method with Hosmer‐Lemeshow test) was also used for dichotomous outcomes related to burnout.

To complement the categorical analysis, stepwise linear regression was conducted for continuous MBI domain scores. While the sample size met the estimated minimum for the primary outcome, the power may still have been insufficient to detect smaller associations in categorical models. Linear regression allowed for the detection of more subtle variations in burnout dimensions and reduced the risk of Type II error. Therefore, the use of both regression types provided a broader and more robust understanding of the relationships between predictors and burnout‐related outcomes. All models used a two‐tailed significance level of *p* < 0.05 and a 95% confidence interval.

Although data were collected at two time points (2020 and 2022), the analyses were conducted treating observations as independent rather than as repeated measures. This decision was based on the substantial loss to follow‐up between waves and the incomplete pairing of participants across time points. Only a subset of respondents could be reliably identified in both phases, and restricting the analyses to fully paired observations would have resulted in a significant reduction in sample size and statistical power. Therefore, each survey wave was analysed as an independent cross‐sectional sample, with the time period included as an explanatory factor when appropriate.

### 2.8 Ethical Considerations

This manuscript is part of a broader, ongoing multicenter study entitled “Work during the COVID‐19 pandemic: impacts on the mental health of nursing workers.” The study was approved on June 30, 2020, by the National Research Ethics Council (approval number 4.152.027) and conducted across four major hospitals for COVID‐19 treatment. Informed consent was obtained via a virtual form, with a copy subsequently sent to participants by email, in accordance with social distancing measures.

## 3 Results

The final sample of the study consisted of 163 participants, who were assessed at two distinct time points. Regarding sociodemographic data, it was observed that the majority of the professionals were female (*n* = 139, 85.3%), with a median age of 41 (37–46) years, self‐identified as white (*n* = 130, 79.8%), married or living with a partner (*n* = 126, 77.3%), and had at least one child (*n* = 120, 73.6%). From the sample, 77 (47.2%) individuals reported having no previous health conditions, although 29 (17.8%) and 28 (17.2%) individuals had cardiovascular and musculoskeletal diseases, respectively.

The occupational characteristics of these professionals are described in Table 1.

**TABLE 1:** Occupational characteristics of the nursing professionals analysed (*N* = 163) (Porto Alegre, RS, Brazil, 2022).

Variables	Frequency
Institution	
HA	19 (11.7%)
HB	24 (14.7%)
HC	86 (52.8%)
HD	34 (20.9%)
Occupation	
Nurse/Registered Nurse	94 (57.7%)
Nursing Technician/Practical Nurse	63 (38.7%)
Nurse Assistant	6 (3.7%)
Experience time (months)*	204 (156–264)
Work contract	
Full time contract	138 (84.7%)
Public servant	25 (15.3%)
Works a second‐job	
Yes	13 (8%)
No	150 (92%)
Managerial position	
Yes	18 (11%)
No	145 (89%)
Work shift	
Morning	68 (41.2%)
Afternoon	41 (25.2%)
Night	46 (28.2%)
On‐call	8 (4.9%)
Department	
Adult Emergency Department	21 (13%)
Adult Intensive Care Unit	20 (12.3%)
Adult Inpatient Unit	47 (29%)
Surgical Center (Operating Room, Post‐Anaesthesia Care Unit, and Sterilisation and Materials Center)	16 (9.9%)
Paediatrics or Neonatology	12 (7.4%)
Outpatient clinic	12 (7.4%)
Other units	61 (37.4%)
Time working in the department*	120 (60–204)
Changed departments during the pandemic	
Yes	56 (34.4%)
No	107 (65.6%)
COVID‐19 ward	
Yes	42 (25.8%)
No	121 (74.2%)
Care for COVID‐19 positive patients	
Yes	144 (88.3%)
No	19 (11.7%)
Increased work demands	
None	1 (0.6%)
Slight	8 (4.9%)
Moderate	25 (15.3%)
Considerable	59 (36.2%)
Severe	70 (42.9%)
Fear of COVID‐19 infection	
None	5 (3.1%)
Slight	19 (11.7%)
Moderate	44 (27%)
Considerable	48 (29.4%)
Severe	47 (28.8%)
Started using medication after the COVID‐19 outbreak	
Yes	44 (27%)
No	119 (73%)
Medical leave due to COVID‐19 suspicion	
Yes	72 (44.2%)
No	91 (55.8%)
Medical leave due to COVID‐19 infection	
Yes	46 (28.2%)
No	117 (71.4%)
Number of days absent due to COVID‐19 suspicion or infection*	0 (0–5)
Medical leave due to other reasons	
Yes	60 (36.8%)
No	103 (63.2%)
Belongs to the COVID‐19 risk group	
Yes	51 (31.3%)
No	112 (68.7%)
Pandemic impact on physical health	
None	4 (2.5%)
Slight	18 (11%)
Moderate	53 (32.5%)
Considerable	52 (31.9%)
Severe	36 (22.1%)
Pandemic impact on mental health	
None	3 (1.8%)
Slight	9 (5.5%)
Moderate	30 (18.4%)
Considerable	59 (36.2%)
Severe	62 (38%)

*Note:* *Variable expressed as median and interquartile range.

The majority of individuals (*n* = 86, 52.8%) were affiliated to Hospital C (HC), working as nurses (*n* = 94, 57.7%). The professionals had a median of 204 months (156–264) of training time, equivalent to about 17 years of professional experience. Most (*n* = 138, 84.7%) were employed under full time contracts, with 150 (92%) of the professionals holding only one job, 68 (41.2%) working in the morning shift, and 18 (11%) occupying managerial positions.

Regarding work departments, 21 (13%) and 20 (12.3%) individuals worked in areas caring for critically ill adult patients, in the Emergency Department and Intensive Care Unit, respectively, while 47 (29.0%) worked in Adult Inpatient Units. Professionals working in other departments and units of their institutions accounted for 37.4% (*n* = 61) of the individuals. The median length of time working in their respective departments was 120 months (60–204), approximately 10 years.

A majority of the professionals (*n* = 121, 74.2%) did not work in units specifically suited for COVID‐19 patients, although the vast majority (*n* = 144, 88.3%) had cared for and treated patients infected by the virus at some point. Moreover, 107 (65.6%) remained in their usual workplace without being reassigned to another department during the pandemic, and 70 (42.9%) reported that their work demands had significantly increased.

In the sample, 5 (3.1%) individuals reported not feeling fear of COVID‐19 infection, although 51 (31.3%) were part of the risk group. In addition, 44.2% (*n* = 72) had been on leave due to COVID‐19 suspicion, and 28.2% (*n* = 46) due to a confirmed infection.

Regarding their physical health during this period, 53 (32.5%), 52 (31.9%), and 36 (22.1%) considered that the pandemic had a “moderate,” “considerable,” or “severe” influence on their physical health, respectively. Furthermore, 59 (36.2%) and 62 (38.0%) reported a “considerable” and “severe” impact of the COVID‐19 pandemic on their mental health, respectively.

Specific data on burnout syndrome (BS) and its domains is summarised in Table 2.

**TABLE 2:** Prevalence of burnout syndrome and scores for the emotional exhaustion, depersonalisation, and low professional accomplishment domains during and after the pandemic in nursing professionals (*N* = 163) (Porto Alegre, RS, Brazil, 2020 and 2022).

Variables	Year		*p*
	2020	2022	
Burnout syndrome			0.493
Yes	15 (9.2%)	12 (7.4%)	
No	148 (90.8%)	151 (92.6%)	
Burnout Inventory domains			
Emotional exhaustion			0.873
Yes	44 (27.0%)	43 (26.4%)	
No	119 (73.0%)	120 (73.6%)	
Depersonalisation			0.477
Yes	46 (28.2%)	41 (25.2%)	
No	117 (71.8%)	122 (74.8%)	
Low professional accomplishment			0.891
Yes	48 (29.4%)	49 (30.1%)	
No	115 (70.6%)	114 (69.9%)	

It was observed that 15 (9.2%) professionals presented with burnout syndrome (BS) in 2020, decreasing to 12 (7.4%) in 2022. Regarding the domains of the inventory, 44 (27.0%) professionals exhibited emotional exhaustion in 2020 and 43 (26.4%) in 2022, while 46 (28.2%) and 41 (25.2%) reported depersonalisation in the same respective periods. Low professional accomplishment, in turn, was identified in 48 (29.4%) and 49 (30.1%) of the workers in 2020 and 2022, respectively. There were no statistically significant differences between the two periods regarding the prevalences evaluated (*p* > 0.05).

Among the professionals who presented with BS in 2020, 11 (6.75%) were nurses, and 4 (2.45%) were nursing technicians. In 2022, 6 (3.68%) were nurses and 6 (3.68%) were nursing technicians.

When comparing the two periods, it is noted that of the 15 (9.2%) individuals affected by BS in 2020, 4 (2.45%) remained with the syndrome until 2022. Additionally, 8 (4.9%) professionals who did not present with BS during the pandemic had developed it in the second assessment.

No significant associations were identified between sociodemographic or occupational variables and BS in the 2020 period (*p* > 0.05). However, in 2022, five variables were statistically associated with the development of BS: medical leave due to other reasons (*p* = 0.012); poor or very poor sleep quality (*p* = 0.014); previous psychiatric disorders (*p* = 0.025); started the use of medications (*p* = 0.033); medical leave due to COVID‐19 suspicion (*p* = 0.046).

Based on the data, logistic regression was performed between the dichotomous outcome of BS and the sociodemographic and occupational variables of nursing professionals during and after the pandemic, as presented in Table 3.

**TABLE 3:** Logistic regression model between the dichotomous outcome of burnout syndrome and the sociodemographic and occupational variables of nursing professionals (*n* = 163) during and after the pandemic (Porto Alegre, RS, Brazil, 2020 and 2022).

Variables	2020	2022
	Prevalence ratio	Prevalence ratio
Pandemic impact on mental health		
Intense	2.63 (1.15–6.01)	NA
None	1	
	*p* = 0.023	
Fear of COVID‐19 infection		
Intense	NA	1.6 (0.99–2.77)
None		1
		*p* = 0.05
Medical leave		
Yes	NA	6.01 (1.25–28.85)
No		1
		*p* = 0.025

Abbreviation: NA = not applicable.

Regarding the logistic regression analysis, in the year 2020, workers who reported an intense impact of the COVID‐19 pandemic on their mental health were 2.6 times more likely to experience burnout syndrome (BS). In 2022, workers who felt intense fear due to exposure to the risk of infection were 1.6 times more vulnerable to the syndrome. Professionals who were presented with medical leave due to other reasons were six times more likely to develop burnout.

Concerning the linear regression between the continuous outcome of BS and the sociodemographic and occupational variables of nursing professionals during and after the pandemic, Table 4 was constructed.

**TABLE 4:** Multivariate linear regression model using the Stepwise method for variables related to burnout syndrome during the pandemic (Porto Alegre, RS, Brazil, 2020).

Variables	*R* ^2^	*β*	DP	Beta	*t*	*p*
Pandemic impact on mental health	0.249	0.510	0.070	0.499	7.303	< 0.001
Care for COVID‐19 patients	0.280	0.546	0.208	0.176	2.621	0.010
Psychiatric disorder	0.305	0.464	0.193	0.162	2.407	0.017
Occupation	0.323	−0.238	0.116	−0.135	−2.054	0.042
*R* = 0.568	*R* ^2^ = 0.323			Standardised *R* ^2^ = 0.306		

Abbreviations: *β*, standardised regression coefficients; Beta, regression coefficients; *R* ^2^, coefficient of determination; SD, standard deviation; *t*, *t*‐test.

In the multivariate linear regression model (Table 4), four variables emerged as predictors, with an explained variance of 30.6%. Analysing the standardised regression coefficients (*β*), the predictors for burnout during the pandemic were: perceiving an impact from the pandemic on mental health, which increased the BS score by 0.51; provided care to COVID‐19 patients, increasing the score by 0.54; previous psychiatric disorder, increasing the score by 0.46; and the occupation of Nursing Technician, which reduced the score by 0.23 (*p* < 0.05).

When analysing Table 5, it is noted that the predictive model of burnout syndrome (BS) after the pandemic was composed of five variables and explains 25.2% of its occurrence. Based on the standardised regression coefficients, the proportional relationship of each variable to BS is observed, as follows: having mental health impacted by the pandemic, which increases the score by 0.34; having a psychiatric disorder, increasing it by 0.64; taken medical leave, adding 0.39; and, respectively, increasing the score by 0.44 and 0.13 units are the variables “holding a managerial position” and “fear of COVID‐19 infection” (*p* < 0.05).

**TABLE 5:** Multivariate linear regression model using the Stepwise method for variables related to burnout syndrome after the pandemic (Porto Alegre, RS, Brazil, 2022).

Variables	*R* ^2^	*β*	SD	Beta	*t*	*p*
Pandemic impact on mental health	0.140	0.340	0.067	0.374	5.099	< 0.001
Psychiatric disorder	0.200	0.646	0.187	0.249	3.446	0.001
Medical leave	0.237	0.392	0.142	0.197	2.768	0.006
Managerial position	0.256	0.442	0.219	0.140	2.016	0.046
Fear of COVID‐19 infection	0.275	0.133	0.065	0.164	2.050	0.042
*R* = 0.525	*R* ^2^ = 0.275			Standardised *R* ^2^ = 0.252		

Abbreviations: *β*, standardised regression coefficients; Beta, regression coefficients; *R* ^2^, coefficient of determination; SD, standard deviation; *t*, *t*‐test.

To examine the relationship between the variables and the domains of BS during the pandemic, a multivariate linear regression analysis was conducted, as presented in Table 6.

**TABLE 6:** Multivariate linear regression model using the Stepwise method for variables related to the domains of emotional exhaustion, depersonalisation, and low professional accomplishment during the pandemic (Porto Alegre, RS, Brazil, 2020).

Variables	*R* ^2^	*β*	SD	Beta	*t*	*p*
Emotional exhaustion						
Pandemic impact on mental health	0.264	4.163	0.548	0.513	7.593	< 0.001
Psychiatric disorder	0.284	3.264	1.546	0.144	2.111	0.036
*R* = 0.533	*R* ^2^ = 0.284			Standardised *R* ^2^ = 0.275		
Depersonalisation						
Pandemic impact on mental health	0.166	1.445	0.255	0.407	5.661	< 0.001
Care for COVID‐19 patients	0.219	2.476	0.753	0.230	3.289	0.001
Psychiatric disorder	0.239	1.437	0.700	0.145	2.054	0.042
*R* = 0.489	*R* ^2^ = 0.239			Standardised *R* ^2^ = 0.225		
Low professional accomplishment						
Pandemic impact on mental health	0.061	−1.229	0.380	−0.247	−3.231	0.001
Occupation	0.096	1.611	0.645	0.188	2.497	0.014
*R* = 0.310	*R* ^2^ = 0.096			Standardised *R* ^2^ = 0.085		

Abbreviations: *β*, standardised regression coefficients; Beta, regression coefficients; *R* ^2^, coefficient of determination; SD, standard deviation; *t*, *t*‐test.

Analysing the table above (Table 6), it is recognised that the predictive model for emotional exhaustion during the pandemic includes two variables, which together explain 27.5% of the occurrence of this dimension among professionals: having mental health impacted by the pandemic, increasing the score by 4.16; previous psychiatric disorder, which increases the score of the evaluated dimension by 3.26.

Regarding depersonalisation, 22.5% of its occurrence during the same period can be explained by the following variables: having mental health impacted by the pandemic, with an increase of 1.44; provided care for COVID‐19, adding 2.47; and previous psychiatric disorder, increasing the score by 1.43.

In turn, professional accomplishment presented the following predictive variables, which explained its variance in 8.5% of cases: having mental health impacted by the pandemic, with a reduction of 1.22; and the Nurse occupation, associated with an increase of 1.61.

The multivariate linear regression model for the relationship between variables and the dimensions of burnout syndrome after the pandemic is described in Table 7.

**TABLE 7:** Multivariate linear regression model using the Stepwise method for variables related to the domains of emotional exhaustion, depersonalisation, and low professional accomplishment after the pandemic (Porto Alegre, RS, Brazil, 2022).

Variables	*R* ^2^	*β*	SD	Beta	*t*	*p*
Emotional exhaustion						
Psychiatric disorder	0.125	7.283	1.525	0.353	4.776	< 0.001
Fear of COVID‐19 infection	0.212	1.891	0.452	0.295	4.187	< 0.001
Medical leave	0.243	2.872	1.115	0.182	2.576	0.011
Pandemic impact on mental health	0.264	1.282	0.591	0.178	2.170	0.032
*R* = 0.515	*R* ^2^ = 0.264			Standardised *R* ^2^ = 0.247		
Depersonalisation						
Pandemic impact on mental health	0.115	1.232	0.269	0.340	4.571	< 0.001
Psychiatric disorder	0.154	2.076	0.768	0.200	2.704	0.008
Managerial position	0.178	1.932	0.916	0.153	2.108	0.037
*R* = 0.421	*R* ^2^ = 0.178			Standardised *R* ^2^ = 0.162		
Low professional accomplishment						
Medical leave	0.040	−2.069	0.802	−0.200	−2.579	0.011
Work department	0.063	0.149	0.075	0.153	1.997	0.048
*R* = 0.252	*R* ^2^ = 0.063			Standardised *R* ^2^ = 0.052		

Abbreviations: *β*, standardised regression coefficients; Beta, regression coefficients; *R* ^2^, coefficient of determination; SD, standard deviation; *t*, *t*‐test.

Upon analysing Table 7, it is noted that the predictive model for emotional exhaustion after the pandemic consisted of four variables, which explained 24.7% of its occurrence: having a psychiatric disorder, increasing the score by 7.28; fear of COVID‐19 infection, increasing it by 1.89; taking medical leave, adding 2.87; and having mental health impacted by the pandemic, increasing the score by 1.28.

Regarding depersonalisation, three predictive variables were identified, explaining 16.2% of the variance. These were: mental health impact by the pandemic, increasing the *z*‐score by 1.23; previous psychiatric disorder, increasing it by 2.07; and holding a managerial position, which increased the score by 1.93.

As for low professional accomplishment, the predictive model explained 5.2% of its occurrence and included two variables: the department in which the professional worked, increasing the score by 0.14, and taking medical leave, associated with a reduction of 2.06. Unlike the previous domains, the latter variable was inversely related to the domain, meaning that the lower its frequency, the greater the occurrence of low professional accomplishment.

## 4 Discussion

The prevalence of professionals with burnout syndrome (BS) in this study was notably high, approaching 10% in 2020 with only a minimal reduction in 2022. The main predictors identified included perceiving an impact of the pandemic on mental health, having a pre‐existing psychiatric disorder, providing care to COVID‐19 patients, and fear of COVID‐19 infection.

The sample profile, predominantly composed of white women with partners and a median age of 41 years, is consistent with findings from studies involving nursing professionals both nationally and internationally (de Magalhães et al. 2022; Olino et al. 2022; Shah et al. 2021). A meta‐analysis indicated that being female, younger, and single increases susceptibility to BS, while younger individuals are more vulnerable due to poorer management of emotional exhaustion and less experience in the role (Ramírez‐Elvira et al. 2021). Gender‐related impacts may also involve a double burden of professional and domestic responsibilities, as highlighted by Soares et al. (2021).

Regarding the pre‐pandemic period, systematic reviews pointed to variations in BS prevalence worldwide, with higher rates in developing countries (Ramírez‐Elvira et al. 2021; Woo et al. 2020). Rates among nurses in developing countries ranged from 15% to 26% depending on socioeconomic context (Hailay et al. 2020; Ohue et al. 2021). A meta‐analysis including 45,539 nurses estimated a global prevalence of 11.2% (Woo et al. 2020), while in Brazil, das Merces et al. (2020) reported 18.3% among nurses. These findings indicate that nursing professionals, particularly in Brazil, were already exposed to considerable levels of BS prior to the pandemic.

During the COVID‐19 pandemic, the literature reports exacerbation of risks due to increased demands and disorganisation of workflows, resulting in physical and psychological overload (Leach et al. 2020; Ampos, Olino, et al. 2023; Kupcova et al. 2023). International studies identified BS symptoms in at least 30% of nurses (Rizzo et al. 2023). Galanis et al. (2021) reported prevalences of 34.1% for emotional exhaustion, 12.6% for depersonalisation, and 15.2% for low professional accomplishment, while in the present study, depersonalisation and low professional accomplishment were proportionally higher than in previous reports. The increase in low professional accomplishment may be associated with the narrative of “heroism” attributed to nurses, which was perceived as symbolic recognition rather than concrete political support (Mohammed et al. 2021).

Contrary to expectations, BS rates and its domains did not significantly differ after the pandemic. One possible explanation lies in the “fourth wave” of overcrowding from patients with acute exacerbations of chronic conditions, which impacted workers' health (Fekadu et al. 2021).

After the pandemic, literature shows that new associations emerged between BS and variables such as poor sleep quality, increased medication use, and medical leave, aligning with findings that link BS to sleep disorders, health deterioration, and increased pharmacological consumption among nurses (Brum et al. 2022; Olino et al. 2022).

Most professionals perceived a moderate to high impact of the pandemic on their mental health, differing from the findings of Centenaro et al. (2023), with a lower rate. This variable also predicted a 2.6 higher chance of BS. Supporting this finding, another regression study identified that poor health status and high frontline work‐related stress are predictors of BS (Wang et al. 2024).

Additionally, absenteeism, especially related to non‐COVID‐19 medical leave, has been described as a contributing factor to BS due to work overload and dissatisfaction (Pinheiro 2024). This may be related to recurrent absenteeism among nursing professionals who are already overburdened, stressed, and unsatisfied with their work.

Lifestyle characteristics such as alcohol consumption, night shift work, lack of physical activity, and perceived work intensification, which are frequent in the current sample, are well established as aggravating factors for BS (Brum et al. 2022; Guerrero et al. 2024). Night shift workers often experience disrupted social relationships due to schedule differences. Furthermore, patients may require more attention during night shifts, which are known for their anxiety‐inducing nature. Night shifts also impact the cortisol cycle, disrupting physiological stress regulation and making professionals more prone to mental disorders (Brum et al. 2022). Alcohol consumption, in turn, is strongly associated with BS. This relationship is understood as alcohol abuse acts as a behavioural coping mechanism. It presents a way to forget about work, as well as an attempt to seek pleasure away from daily responsibilities due to poor working conditions (Olino et al. 2022). Moreover, regular physical activity, which was low in the current sample, is recognised as a protective factor against BS (Guerrero et al. 2024), as it promotes the release of endorphins, improves mood, and reduces stress.

Although one‐third of the sample belonged to the COVID‐19 high‐risk group, more than half of the professionals reported moderate to intense fear of infection. This fear can be explained by several factors, including lack of knowledge about the virus, insufficient protective equipment, and the constant risk of infection inherent to nursing practice (Podgorica et al. 2022; Ampos, Vecchia, et al. 2023).

In both assessments, having a pre‐existing psychiatric disorder and experiencing the impact of the pandemic on mental health were predictors of BS and emotional exhaustion, in line with the literature describing the impact of the healthcare crisis on workers' mental health (Kupcova et al. 2023; Olino et al. 2022). Prior psychiatric disorders are associated with higher BS prevalence, as they make professionals more vulnerable to the syndrome's development due to their already active symptoms (Woo et al. 2020). Moreover, in 2020, a report by the World Health Organization indicated a 25% increase in the global prevalence of anxiety and depression (WHO 2022). Therefore, it is expected that BS prevalence would be substantially high, and that these would be significant predictive variables.

Furthermore, during the first period of this cohort, both caring for COVID‐19 patients and the current occupation presented as significant variables in the BS predictive model. The variable related to patient care seems to be linked to fear of infection, especially since, at the time, the virus was still unknown and there was no vaccine or treatment available. Supporting this, a study executed during the pandemic identified differences between professionals working in COVID‐dedicated units and non‐COVID units, finding higher levels of anxiety, fear, and minor psychological disorders among those in dedicated units, as well as higher emotional exhaustion rates (Ampos, Vecchia, et al. 2023; Galanis et al. 2021). In Brazil, a mixed‐methods study by Ampos, Vecchia, et al. (2023) revealed that, even among professionals not assigned to dedicated units, 83.3% had contact with and/or provided care for COVID‐19 patients, which helps to explain the fear of infection reported by workers.

Regarding the predictor “occupation”, nurses were more likely to score higher for BS and its domains than nursing technicians. While nursing technicians' duties traditionally require more physical effort and direct patient contact (Ferreira and de Lucca 2015), Brazilian nurses' roles involve greater responsibility, decision‐making, management of the unit, the team, and patient care (de Magalhães et al. 2022).

These responsibilities translate into high BS rates in both professional categories. However, while the prevalence among technicians was 5.9% prior to the pandemic (Ferreira and de Lucca 2015), it was 18.3% among nurses (das Merces et al. 2020). This gap narrowed during the pandemic, with prevalence increasing in both groups. The literature shows considerable heterogeneity, as different contexts yielded different outcomes for professionals; nonetheless, in a similar sample, prevalence remained higher among nurses (17.4%) than technicians (9.5%) (de Magalhães et al. 2022).

After the pandemic, intense fear of infection and recurrent medical leave were identified as predictors in the logistic regression model. These two variables were also found to predict emotional exhaustion after the pandemic in the linear regression model. Both findings are consistent with predictors identified in the literature (Wang et al. 2024).

In respect of working in a managerial position as a predictor, Wei et al. (2019) indicated that leaders tend to exhibit greater resilience and better coping abilities for burnout, thus presenting lower BS rates. In the present study, workers in this position presented higher rates of BS, with depersonalisation being a major symptom. This novel finding contrasts with the current literature and reflects the intense decision‐making demands of working as a manager in healthcare.

Regarding the identification of fear as a predictor, one can infer that, around 2022, new virus variants had emerged, raising concerns about whether vaccines would provide adequate protection. Additionally, fear of infection was intense among professionals regardless of whether they worked in COVID‐dedicated units. Most professionals in this study were not reassigned to specific COVID‐19 units. Nevertheless, consistently with Ampos, Vecchia, et al. (2023), a major part of the present sample reported fear of exposure to COVID‐19. Thus, it would be important to further investigate post‐pandemic fear in the future, preferably through a qualitative approach, to understand how the fear of contamination persists among professionals even after the peak of the health crisis, and how this influences mental health and work processes.

Finally, it is important to highlight that burnout syndrome is a complex, multifactorial occupational phenomenon influenced by individual, institutional, and contextual determinants. The findings of this study contribute to the body of evidence on the impact of the COVID‐19 pandemic on the mental health of nursing professionals. Findings suggest that the long‐term effects of occupational stress still remain, even after the end of the public health emergency. The identified predictors should be considered for developing institutional policies aimed at promoting the mental health of healthcare workers.

Moreover, the results underscore the importance of continuous and sustained preventive strategies, such as strengthening support networks in the workplace, ensuring adequate working conditions, implementing mental health promotion programs, and professional recognition—all of which should extend beyond times of crisis. The persistence of burnout symptoms after the pandemic indicates that addressing professional exhaustion in nursing should be a management priority not only in emergency scenarios but as an integral part of the daily operation of healthcare services.

## 5 Conclusions

It was observed that the majority of professionals were middle‐aged white women, married, and with at least one child. High prevalences of burnout syndrome (BS) were noted among the workers, with slightly lower scores in 2022. The workers presented distinct predictive factors for BS and its dimensions, the main ones being having previous psychiatric disorders, perceiving a strong impact of the pandemic on mental health, feeling fear of COVID‐19 infection, and having taken multiple non‐COVID‐19 medical leaves.

Based on the variables identified as predictors of the development of BS, this study reinforces the identification of harmful effects on workers' mental health. The results highlight the need for further research to assess whether the levels of professionals with BS will persist over the years, following the resolution of overcrowding caused by the backlog of patients after the pandemic.

One limitation of the study is the healthy worker effect bias; that is, those who remained active in the labor market during the first data collection period, preventing the inclusion of data from professionals who were on leave due to health issues during the two‐year follow‐up period.

It is essential to invest in the ability of health services to recognise the signs and symptoms of BS, with an emphasis on the variables that present higher risk for psychological illness, aiming to prevent these problems whenever possible or to provide emotional and psychological support to affected workers. Thus, the development of institutional actions and interdisciplinary public policies focused on mental health is urgent. Similarly, organisational management needs to be committed to implementing improvements that involve workers in the process of change in the work environment, with a focus on practices and training related to the development of resilience, in order to protect mental health.

The pandemic was a difficult and traumatic period for nursing workers, bringing unprecedented challenges and stressors, which may have long‐term consequences for the mental health of these professionals. More studies are still needed, especially those that propose interventions to improve the health of these workers.

## 6 Relevance for Clinical Practice

The findings underscore the urgent need for institutions to adopt proactive strategies that protect and promote the mental health of nursing professionals, particularly in post‐crisis contexts. Identifying predictors of burnout syndrome—such as previous psychiatric disorders, perceived impact of the pandemic on mental health, fear of infection, and repeated medical leaves—enables early risk recognition and the implementation of targeted interventions.

For managers and leaders, incorporating these predictors into occupational health protocols, systematic mental health screening, and evidence‐based support programs can enhance workforce resilience, reduce absenteeism, and mitigate professional attrition. At the policy level, the results provide critical evidence to inform the development of public health initiatives aimed at ensuring adequate working conditions, strengthening staff retention, and embedding mental health promotion as a structural component of healthcare systems. Ultimately, translating these insights into managerial practices and health policies can contribute to safer care delivery, improved patient outcomes, and a more sustainable nursing workforce.

## Author Contributions

Made substantial contributions to conception and design, or acquisition of data, or analysis and interpretation of data: Miguel Lucas Silva da Paixão, Daiane Dal Pai, Tânia Solange Bosi de Souza Magnago, Fábio Fernandes Dantas Filho, Silvia Cristina Garcia Carvalho, Gabriel Fernandes Gonçalves, Luciana Olino, Juliana Petri Tavares. Involved in drafting the manuscript or revising it critically for important intellectual content: Miguel Lucas Silva da Paixão, Daiane Dal Pai, Tânia Solange Bosi de Souza Magnago, Fábio Fernandes Dantas Filho, Silvia Cristina Garcia Carvalho, Gabriel Fernandes Gonçalves, Luciana Olino, Juliana Petri Tavares. Given final approval of the version to be published. Each author should have participated sufficiently in the work to take public responsibility for appropriate portions of the content: Miguel Lucas Silva da Paixão, Daiane Dal Pai, Tânia Solange Bosi de Souza Magnago, Fábio Fernandes Dantas Filho, Silvia Cristina Garcia Carvalho, Gabriel Fernandes Gonçalves, Luciana Olino, Juliana Petri Tavares. Agreed to be accountable for all aspects of the work in ensuring that questions related to the accuracy or integrity of any part of the work are appropriately investigated and resolved: Miguel Lucas Silva da Paixão, Daiane Dal Pai, Tânia Solange Bosi de Souza Magnago, Fábio Fernandes Dantas Filho, Silvia Cristina Garcia Carvalho, Gabriel Fernandes Gonçalves, Luciana Olino, Juliana Petri Tavares.

## Funding

Funding was provided by the Hospital de Clinicas de Porto Alegre (ROR: https://ror.org/010we4y38) and by the Brazilian National Council for Scientific and Technological Development (CNPq) through Master's scholarship.

## Disclosure

The authors have checked to make sure that our submission conforms as applicable to the Journal's statistical guidelines described. The statistics were checked prior to submission by an expert statistician (Wagner Lara Machado, wag.lm.psico@gmail.com). The authors affirm that the methods used in the data analyses are suitably applied to their data within their study design and context, and the statistical findings have been implemented and interpreted correctly. The authors agree to take responsibility for ensuring that the choice of statistical approach is appropriate and is conducted and interpreted correctly as a condition to submit to the Journal.

## Ethics Statement

This study was conducted in accordance with ethical standards and approved by the institution's ethics committee.

## Consent

All participants provided informed consent through a signed Informed Consent Form, in accordance with applicable ethical guidelines.

## Conflicts of Interest

The authors declare no conflicts of interest.

## Data Availability

The data that support the findings of this study are available from the corresponding author, Miguel Lucas Silva da Paixão, upon reasonable request.
